# Eco-anxiety’s dual role in adherence to the Mediterranean and DASH diets: the mediating roles of depression, anxiety, and stress

**DOI:** 10.3389/fpsyg.2025.1701068

**Published:** 2026-01-12

**Authors:** Büşra Başar Gökcen, Rabia Büşra Işın, Duygu Ağagündüz, Tuba Esatbeyoglu, Fatih Ozogul

**Affiliations:** 1 Department of Nutrition and Dietetics, Fethiye Faculty of Health Sciences, Muğla Sıtkı Koçman University, Muğla, Türkiye; 2 Department of Nutrition and Dietetics, Faculty of Health Sciences, Gazi University, Ankara, Türkiye; 3 Department of Molecular Food Chemistry and Food Development, Institute of Food and One Health, Gottfried Wilhem Leibniz University Hannover, Hannover, Germany; 4 Department of Seafood Processing Technology, Faculty of Fisheries, Çukurova University, Adana, Türkiye

**Keywords:** Mediterranean Diet, Dietary Approaches to Stop Hypertension, eco-anxiety, depression, anxiety, stress

## Abstract

**Background:**

The Mediterranean and the Dietary Approaches to Stop Hypertension (DASH) diet are recognized for their cardiometabolic and preventive health benefits. However, the psychological and environmental determinants of adherence to these dietary patterns remain underexplored. The present study examined the direct and indirect effects of eco-anxiety, eco-awareness, and psychological distress on adherence to these healthy and sustainable diets.

**Methods:**

A cross-sectional online survey was conducted among 1,028 Turkish adults aged 18–65 years. Data were collected using validated instruments, including the Mediterranean Diet Adherence Screener (MEDAS), DASH-Questionnaire (DASH-Q), Depression Anxiety Stress Scale-21 (DASS-21), Hogg Eco-Anxiety Scale (HEAS), and the Awareness Scale for Reducing Ecological Footprint (ASREF). Statistical analyses included correlation tests, linear regression, and mediation models (PROCESS macro).

**Results:**

Linear regression analyses indicated that eco-anxiety significantly predicted higher adherence to both MEDAS [B = 0.329, 95% CI (0.101–0.557)] and DASH-Q [B = 1.409, 95% CI (0.234–2.585)], while eco-awareness predicted only DASH-Q [B = 2.289, 95% CI (1.719–2.859)]. Psychological models revealed that eco-anxiety strongly predicted depression, anxiety, and stress. Conversely, higher MEDAS [B = −0.157, 95% CI (0.279–0.034)] and DASH-Q scores [B = −0.027, 95% CI (0.051–0.003)] were associated with lower depression, while both also predicted lower anxiety. Mediation analyses confirmed significant negative indirect effects of depression and anxiety in the association between eco-anxiety and both MEDAS and DASH-Q, while stress mediated only the relationship between eco-anxiety and MEDAS. Notably, in all models the direct paths from eco-anxiety to dietary adherence remained positive.

**Conclusion:**

The results indicate a potential dual pattern in how eco-anxiety relates to dietary adherence. While eco-anxiety shows a positive association with adherence to healthy and sustainable diets, its connection with psychological distress is associated with lower adherence. Although these findings do not imply causality, they underscore the importance of considering both environmental concerns and psychological distress when examining determinants of dietary adherence.

## 1 Introduction

Climate change has become an increasingly severe global crisis, posing substantial risks to food security, housing, and public health (Qi et al., 2022). Growing recognition of its human-driven nature has led to the emergence of eco-anxiety, a set of emotional responses to environmental degradation, particularly climate change (Mento et al., 2023; Brophy et al., 2023). Eco-anxiety encompasses feelings of worry, fear, and sadness related to ecological threats and reflects rising concern over the multifaceted consequences of the climate crisis. Although no single definition exists, it is commonly described as distress triggered by awareness of environmental change, evoking feelings of helplessness or anxiety (Kankawale and Niedzwiedz, 2023; Usher et al., 2019; Gayathri et al., 2025). Greater exposure to information about the climate crisis may heighten perceived threat and elicit emotional responses such as stress, fear, hopelessness, and guilt (Memiç-İnan et al., 2025). Eco-anxiety has been linked to adverse psychological outcomes, including depression, generalized anxiety, stress, and sleep disturbances, with higher levels reported among younger individuals, women, and climate-vulnerable communities (Boluda-Verdú et al., 2022). On the other hand, eco-anxiety may function not only as a source of psychological distress but also as a motivator that drives proactive, environmentally protective behaviors, including shifts toward sustainable dietary practices (Kerse and Kerse, 2025).

Adopting sustainable dietary patterns is increasingly recognized as a key strategy for mitigating the adverse health and environmental consequences of climate change (Kabasakal-Cetin, 2023). According to the World Health Organization, sustainable healthy diets are those that support overall health, exert low environmental impact, and remain accessible, affordable, safe, and culturally acceptable (Food and Agriculture Organization of the United Nations and World Health Organization, 2019). The Mediterranean Diet is one of the most prominent examples of sustainable dietary approaches, owing to its low environmental impact, deep-rooted socio-cultural food traditions, contributions to local economies, and well-established health benefits (Portugal-Nunes et al., 2021). There is a strong association between environmentally friendly food choices and adherence to the Mediterranean Diet, which further underscores its distinguished position among healthy and environmentally conscious nutritional models (Yassıbaş and Bölükbaşı, 2023).

The Dietary Approaches to Stop Hypertension (DASH) diet, classified among healthy dietary models, is recognized for supporting both human and environmental health (Kling et al., 2023). Originally developed for the prevention and management of hypertension, the DASH Diet has been associated with improved public health outcomes and reductions in greenhouse gas emissions linked to dietary practices (Monsivais et al., 2015). Although increases in fruit and whole grain consumption and reductions in red and processed meat intake are associated with lower greenhouse gas emissions, definitive conclusions cannot be drawn regarding the sustainability of all components of the DASH dietary model (Monsivais et al., 2015; Kling et al., 2023). Notably, Conrad et al. (2025) reported that, across seven sustainability indicators, the Mediterranean Diet demonstrated more favorable environmental outcomes compared to the DASH Diet.

Despite its well-documented benefits for both human and environmental health, adherence to the Mediterranean Diet has been declining across Mediterranean populations (Guasch-Ferré and Willett, 2021; Soltani et al., 2020; Quarta et al., 2021). Evidence from five Mediterranean and two non-Mediterranean countries indicates that a considerable proportion of adults exhibit low adherence to this dietary pattern (Quarta et al., 2021), a trend also observed in studies conducted in Türkiye (Yassıbaş and Bölükbaşı, 2023; Yıldırım and Kaya Çebioğlu, 2021; Batu et al., 2025). A recent systematic review covering 12 countries identified several key barriers to Mediterranean Diet adherence, including financial constraints, limited knowledge, lifestyle and health challenges, socio-cultural and motivational factors, limited accessibility, hedonic preferences, and demographic characteristics (Tsofliou et al., 2021). Similarly, several barriers to adherence to the DASH Diet have been identified, including the high cost of healthful foods, limited familiarity with recommended serving sizes, inadequate cooking skills, and negative influence from household members. In contrast, facilitators of adherence include access to healthful foods, living with supportive household members, and possessing willpower as well as internal or external motivation for dietary change (Tyson et al., 2023).

Taken together, these findings highlight the need to better understand the psychological and environmental determinants that influence adherence to healthy and sustainable dietary models such as the Mediterranean and DASH diets (Fatemi et al., 2025). While considerable efforts have been made to understand the cognitive and motivational determinants of sustainable eating—primarily through frameworks such as the Theory of Planned Behavior and the Theory of Behavioral Choice—much less attention has been given to clinically relevant psychological variables that may hinder or facilitate dietary adherence. However, psychological distress—an important determinant in the translation of sustainable dietary intentions into actual behavior—has not been examined in a comprehensive manner within the existing literature (Lo Dato et al., 2025). Particularly, the dual role of eco-anxiety—acting as both a motivator for healthier dietary adherence and a risk factor through psychological distress—remains insufficiently explored in the literature. This underscores the need for more comprehensive models that integrate environmental concerns and psychological processes to better explain diet-related behaviors. The aim of this study is to investigate the environmental and psychological distress determinants of adherence to the Mediterranean and DASH diets, with a particular focus on testing the direct and indirect effects of eco-anxiety and evaluating the mediating role of psychological distress factors in these associations.

## 2 Materials and methods

### 2.1 Study design and population

This cross-sectional study was carried out in Turkey, and data were collected between June and November 2024 using a self-administered digital questionnaire (Google Forms®) distributed via dedicated links and QR codes. Participation was voluntary, and all participants provided electronic informed consent prior to enrollment.

Considering the Turkish adult population of approximately 55 million, with a 95% confidence level, 5% margin of error, and *p* = 0.5, the minimum required sample size was calculated as 385 participants using the finite population correction formula. To enhance representativeness, purposive and snowball sampling strategies were employed through online dissemination on social media platforms and personal networks. Data collection continued until no additional responses were recorded for seven consecutive days, which was taken as evidence that the response rate had plateaued. At that point, a total of 1,041 individuals had completed the questionnaire.

During data cleaning, participants outside the 18–65 age range, those with implausible anthropometric values (height and weight), and individuals with incomplete or inconsistent responses were excluded. Specifically, height values below 120 cm or above 220 cm, and weight values below 30 kg or above 200 kg, were considered implausible. To further ensure attentiveness and data validity, attention-check questions (e.g., ‘Please select *sometimes* for this item’) were embedded in the questionnaire, and participants failing these checks were also excluded. As a result, the final analytic sample consisted of 1,028 participants.

This study was approved by the Muğla Sıtkı Koçman University Medical and Health Sciences Ethics Committee on March 6, 2024 (Protocol No: 230166-40). All procedures were conducted in accordance with the Declaration of Helsinki.

### 2.2 Data collection procedures and tools

The questionnaire was structured into four main sections, aiming to capture sociodemographic, dietary, psychological, and environmental aspects of the participants:

#### 2.2.1 Sociodemographic and anthropometric data

Age, gender, marital status, weight, height and body mass index (BMI), smoking and alcohol habits, and presence of chronic diseases. Height and weight were obtained through self-report by participants, which may introduce potential reporting bias.

#### 2.2.2 Adherence to a sustainable-healthy diet

Adherence to the Mediterranean diet was assessed using the 14-item Mediterranean Diet Adherence Screener (MEDAS), originally developed by Martínez-González et al. (2012) within the PREDIMED trial. The Turkish validity and reliability study was conducted by Pehlivanoğlu et al. (2020). Scores range from 0 to 14, with higher scores indicating greater adherence (Pehlivanoğlu et al., 2020). Adherence to the DASH dietary pattern was measured with the Dietary Approaches to Stop Hypertension Questionnaire (DASH-Q), developed by Warren-Findlow et al. (2017) and validated in Turkish by Çetin and Baş (2020). This 11-item tool uses a 7-day recall format. Scores range from 0 to 77, with higher scores indicating greater adherence.

#### 2.2.3 Psychological distress

The Depression Anxiety Stress Scale-21 (DASS-21) was developed by Lovibond and Lovibond (1995) and initially consisted of 42 items, divided into three subscales: depression, anxiety, and stress. The Turkish adaptation of the 21-item short form, along with its validity and reliability study, was conducted by Sariçam (2018). It is a 4-point Likert-type scale with response options ranging from never (0 points), sometimes (1 point), frequently (2 points), to always (3 points). In this study, raw subscale scores of the DASS-21 were used, without multiplying by two.

#### 2.2.4 Environmental sensitivity

The Hogg Eco-Anxiety Scale (HEAS), developed by Hogg et al. (2021) and validated in Turkish by Uzun et al. (2022), was employed to assess eco-anxiety. This 13-item instrument includes four subscales: affective symptoms, rumination, behavioral symptoms, and anxiety about personal impact. Items are rated on a 4-point Likert scale (0 = never to 3 = almost always), with higher scores indicating elevated eco-anxiety (Hogg et al., 2021; Uzun et al., 2022). In addition, the Awareness Scale for Reducing Ecological Footprint (ASREF), developed by Tekindal et al. (2021), was used to measure eco-awareness across six domains (energy, laws, recycling, transportation, water, and nutrition). It consists of 27 items rated on a 5-point Likert scale (1 = strongly disagree to 5 = strongly agree). Higher scores indicate greater awareness of ecological footprint reduction (Tekindal et al., 2021).

### 2.3 Statistical analysis

All statistical analyses were performed using IBM SPSS Statistics for Windows, Version 30.0 (IBM Corp., Armonk, NY, USA) and PROCESS macro version 4.2 developed by Andrew F. Hayes. The significance level was set at *p* < 0.05 (two-tailed). Distributional properties of continuous variables were assessed using the Shapiro–Wilk test, histograms, Q–Q plots, and skewness/kurtosis indices. Descriptive statistics (mean, standard deviation, and median) were calculated for all study variables. Pearson’s correlation coefficients were computed to examine bivariate associations among the study variables. Correlation analyses were conducted for descriptive purposes; therefore, no correction for multiple testing was applied. Prior to main analyses, data were examined for normality, linearity, and multicollinearity assumptions. Variance inflation factor (VIF) and tolerance values were inspected to assess multicollinearity; all values remained within acceptable ranges (VIF < 5; tolerance > 0.70).

Linear regression analyses were conducted to identify predictors of dietary adherence, with continuous MEDAS and DASH-Q scores entered as dependent variables. Independent variables included age and BMI (continuous), gender (categorical: 1 = female, 2 = male), eco-anxiety, and eco-awareness. For each model, both unstandardized coefficients (B) and standardized effect sizes (β), along with 95% confidence intervals (CI), *p*-values, F-change, and R^2^ values, were reported. When depression, anxiety, and stress were added to the models, high Variance Inflation Factor (VIF) values indicated multicollinearity; therefore, these variables were examined in separate regression models, under which dietary adherence was also evaluated.

Finally, mediation analyses were performed using PROCESS macro (Model 4) to test whether depression, anxiety, and stress mediated the association between eco-anxiety and dietary adherence (MEDAS, DASH-Q). Three separate models were specified: Model 1 (mediator = depression), Model 2 (mediator = anxiety), and Model 3 (mediator = stress). Both direct (X → Y) and indirect (X → M → Y) pathways were estimated, with bias-corrected bootstrapping (5,000 resamples) used to assess the significance of indirect effects. All models were adjusted for age, gender, and BMI.

## 3 Results

Participants had a mean age of 35.17 years (SD = 12.70; median = 32; range = 18–65). The mean BMI was 24.58 kg/m^2^ (SD = 4.33; median = 24.3; range = 14.7–46.7). According to BMI classification, 4.8% were underweight, 53.1% were normal weight, 30.5% were overweight, and 11.6% were obese. The sample was 54.1% female (n = 556) and 45.9% male (n = 472). Regarding lifestyle characteristics, 32.1% (n = 330) of participants smoked, 23.2% (n = 239) consumed alcohol, and 17.9% (n = 184) reported having a chronic disease. For Mediterranean diet adherence, 23.9% (n = 246) reported low adherence, 60.1% (n = 618) moderate adherence, and 16.0% (n = 164) high adherence.

Table 1 presents descriptive statistics and intercorrelations among the study variables. MEDAS was correlated with DASH-Q (r = 0.268), eco-anxiety (HEAS) (r = 0.084), eco-awareness (ASREF) (r = 0.094), depression (r = −0.077), and anxiety (r = −0.073). DASH-Q was correlated with eco-anxiety (r = 0.122) and eco-awareness (r = 0.249).

**Table 1:** Descriptive statistics and intercorrelations among study variables.

Variables	Mean ±SD (Median)	Correlation Coefficients (r) and *p* values											
		2		3		4		5		6		7	
		r	*p*	r	*p*	r	*p*	r	*p*	r	*p*	r	*p*
MEDAS	7.1 ± 2.2 (7.0)	0.268	**<0.001**	0.084	**0.007**	0.094	**0.003**	−0.077	**0.014**	−0.073	**0.019**	−0.043	0.172
DASH-Q	27.7 ± 11.7 (26.0)			0.122	**<0.001**	0.249	**<0.001**	−0.004	0.910	−0.027	0.390	0.033	0.288
HEAS	0.9 ± 0.6 (0.9)					−0.002	0.939	0.524	**<0.001**	0.545	**<0.001**	0.541	**<0.001**
ASREF	3.5 ± 1.2 (3.9)							−0.057	0.066	−0.101	**0.001**	−0.017	0.596
Depression	6.1 ± 5.0 (6.0)									0.824	**<0.001**	0.847	**<0.001**
Anxiety	5.3 ± 4.6 (5.0)											0.842	**<0.001**
Stress	6.5 ± 4.7 (6.0)											-	

Pearson correlation coefficients (r) and exact p-values are reported for all variable pairs. Correlation analyses were conducted for descriptive purposes; therefore, no correction for multiple testing was applied. MEDAS: Mediterranean Diet Adherence Screener, DASH-Q: Dietary Approaches to Stop Hypertension Questionnaire, HEAS: Hogg Eco-Anxiety Scale, ASREF: Awareness Scale for Reducing Ecological Footprint. Values presented in bold indicate statistically significant results (*p* < 0.05).

Linear regression analyses for MEDAS and DASH-Q scores are shown in Table 2. For MEDAS, age was positively associated with adherence (B = 0.051, 95% CI [0.040, 0.063]). BMI was not statistically significant. Gender was associated with MEDAS scores (B = −0.414, 95% CI [−0.686, −0.142]). Eco-anxiety showed a positive association with MEDAS (B = 0.329, 95% CI [0.101, 0.557]). Eco-awareness was not statistically significant.

**Table 2:** Linear regression models predicting MEDAS and DASH-Q scores.

Predictors	MEDAS Score					DASH-Q Score				
	B	β	95% CI		*p*	B	β	95% CI		*p*
Age	0.051	0.292	0.040	0.063	**<0.001**	−0.163	0.031	−0.223	−0.103	**<0.001**
BMI	−0.026	−0.050	−0.059	0.008	0.132	−0.220	0.088	−0.392	−0.047	**0.013**
Gender	−0.414	−0.093	−0.686	−0.142	**0.003**	−0.417	0.718	−1.826	0.991	0.561
Eco-anxiety	0.329	0.084	0.101	0.557	**0.005**	1.409	0.599	0.234	2.585	**0.019**
Eco-awareness	−0.051	−0.027	−0.165	0.063	0.380	2.289	0.291	1.719	2.859	**<0.001**
MEDAS or DASH-Q	0.054	0.285	0.043	0.066	**<0.001**	1.437	0.155	1.133	1.741	**<0.001**
Model Summary	R^2^: 0.146 and F-change: 29.043					R^2^: 0.183 and F-change: 38.201				

The gender variable was defined as categorical, with coding set as 1 for female and 2 for male. In the MEDAS model, DASH-Q was included as the diet-related predictor. In the DASH-Q model, MEDAS served as the predictor. For the MEDAS model, the VIF values ranged between 1.042 and 1.325, and the tolerance values ranged between 0.754 and 0.960. For the DASH-Q model, the VIF values ranged between 1.044 and 1.380, and the tolerance values ranged between 0.725 and 0.958. MEDAS: Mediterranean Diet Adherence Screener, DASH-Q: Dietary Approaches to Stop Hypertension Questionnaire, HEAS: Hogg Eco-Anxiety Scale, ASREF: Awareness Scale for Reducing Ecological Footprint, BMI: Body Mass Index. Values presented in bold indicate statistically significant results (*p* < 0.05).

For DASH-Q, age showed a negative association (B = −0.163, 95% CI [−0.223, −0.103]), and BMI was negatively associated with scores (B = −0.220, 95% CI [−0.392, −0.047]). Gender was not statistically significant. Eco-anxiety (B = 1.409, 95% CI [0.234, 2.585]) and eco-awareness (B = 2.289, 95% CI [1.719, 2.859]) both showed positive associations with DASH-Q. Associations between MEDAS and DASH-Q were also identified in both directions.

Regression analyses for DASS-21 subscales are summarized in Table 3. Eco-anxiety was positively associated with depression, anxiety, and stress across models. Eco-awareness was not significantly associated with depression or stress and showed a small negative association with anxiety. MEDAS scores were negatively associated with depression (B = −0.157, *p* = 0.012), anxiety (B = −0.124, *p* = 0.030), and stress (B = −0.122, *p* = 0.040). DASH-Q scores were negatively associated with depression (B = −0.027, *p* = 0.026) and anxiety (B = −0.033, *p* = 0.003), but not with stress.

**Table 3:** Linear regression models predicting DASS-21 depression, anxiety, and stress scores.

Predictors	Depression score					Anxiety score					Stress score				
	B	β	95% CI		*p*	B	β	95% CI		*p*	B	β	95% CI		*p*
Age	−0.067	−0.173	−0.091	−0.044	**<0.001**	−0.064	−0.177	−0.086	−0.043	**<0.001**	−0.048	−0.129	−0.070	−0.025	**<0.001**
BMI	0.055	0.048	−0.012	0.122	0.108	0.059	0.055	−0.002	0.119	0.060	0.051	0.047	−0.012	0.115	0.114
Gender	−0.299	−0.030	−0.844	0.247	0.283	−0.357	−0.039	−0.854	0.139	0.158	−0.465	−0.049	−0.985	0.054	0.079
Eco-anxiety	4.472	0.510	4.016	4.929	**<0.001**	4.341	0.532	3.926	4.756	**<0.001**	4.409	0.528	3.974	4.844	**<0.001**
Eco-awareness	−0.077	−0.018	−0.304	0.151	0.508	−0.232	−0.059	−0.439	−0.025	**0.028**	0.025	0.006	−0.191	0.242	0.818
MEDAS	−0.157	−0.070	−0.279	−0.034	**0.012**	−0.124	−0.060	−0.235	−0.012	**0.030**	−0.122	−0.058	−0.239	−0.006	**0.040**
DASH-Q	−0.027	−0.064	−0.051	−0.003	**0.026**	−0.033	−0.083	−0.054	−0.011	**0.003**	−0.014	−0.035	−0.037	0.008	0.216
Model Summary	R^2^: 0.316 and F-change: 67.470					R^2^: 0.348 and F-change: 77.672					R^2^: 0.317 and F-change: 67.517				

The gender variable was defined as categorical, with coding set as 1 for female and 2 for male. For each model including anxiety, depression, and stress separately, the VIF values ranged between 1.050 and 1.418, and the tolerance values ranged between 0.705 and 0.952. MEDAS, Mediterranean Diet Adherence Screener; DASH-Q, Dietary Approaches to Stop Hypertension Questionnaire; HEAS, Hogg Eco-Anxiety Scale; ASREF, Awareness Scale for Reducing Ecological Footprint; BMI, Body Mass Index. Values presented in bold indicate statistically significant results (*p* < 0.05).

**Figure 1:**
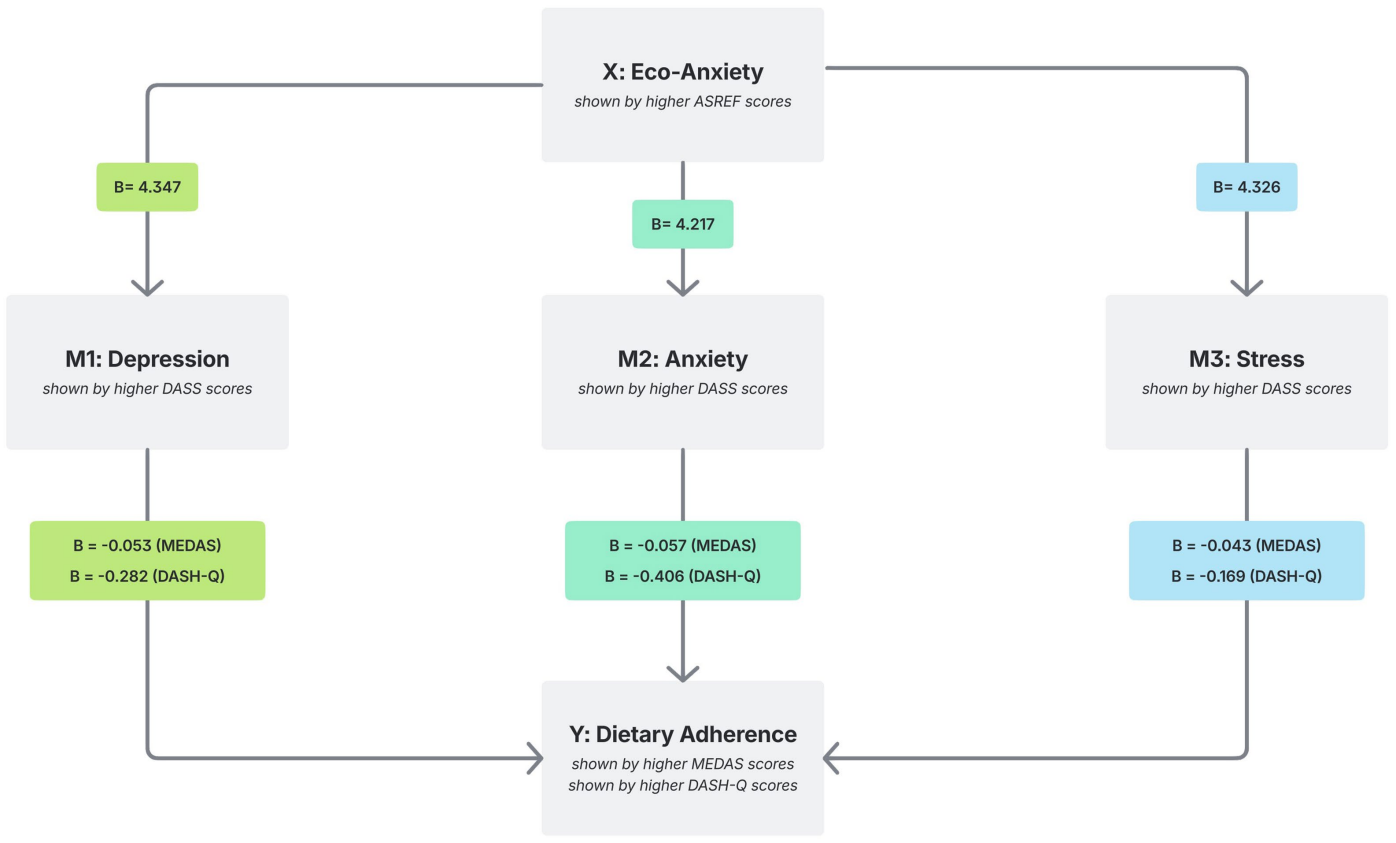
Mediation effects of depression, anxiety, and stress on the relationship between eco-anxiety and dietary adherence. In the pathways from eco-anxiety (X) to healthy dietary adherence (Y: MEDAS, DASH), three separate mediators were tested: Model 1: depression (M1), Model 2: anxiety (M2), and Model 3: stress (M3). In each model, the paths from X to M (X → M) and from M to Y (M → Y) were examined, together with the direct effect of X on Y (X → Y) and the indirect effect through the mediator (X → M → Y). All analyses were adjusted for age, gender, and BMI. MEDAS, Mediterranean Diet Adherence Screener; DASH-Q, Dietary Approaches to Stop Hypertension Questionnaire; HEAS, Hogg Eco-Anxiety Scale; ASREF, Awareness Scale for Reducing Ecological Footprint; BMI, Body Mass Index.

Mediation analyses examining indirect associations are presented in Figure 1. In Model 1, eco-anxiety was positively associated with depression, and depression showed negative associations with both MEDAS and DASH-Q. In Model 2, eco-anxiety was positively associated with anxiety, which also showed negative associations with MEDAS and DASH-Q. In Model 3, eco-anxiety was positively associated with stress. Stress showed a negative association with MEDAS but was not significantly associated with DASH-Q. Significant indirect paths were identified for MEDAS and DASH-Q across models, except for the stress model in relation to DASH-Q. Direct associations remained statistically significant in all models.

## 4 Discussion

Despite the growing interest in sustainable dietary approaches in recent years, adherence to healthy and sustainable dietary patterns such as the Mediterranean and DASH diets remains low at the population level (Kling et al., 2023; Lorca-Camara et al., 2024; Fanzo and Davis, 2019; Portugal-Nunes et al., 2023; Fanzo et al., 2022). This highlights the need for a detailed examination of the psychosocial and environmental factors that shape individual dietary behaviors. The present study aimed to evaluate the direct and indirect associations between eco-anxiety and adherence to the Mediterranean and DASH diets.

The descriptive and correlational findings of this study provide a comprehensive overview of how ecological and psychological factors relate to adherence to healthy dietary patterns. Both MEDAS and DASH-Q scores showed significant positive associations with eco-anxiety and eco-awareness, suggesting that individuals with greater environmental concern and awareness tend to report higher adherence to the Mediterranean and DASH diets. In contrast, negative associations were observed with depression and anxiety, indicating that higher levels of psychological distress were linked to lower engagement in healthy dietary behaviors. Consistent with these correlations, the regression analyses demonstrated that eco-anxiety and eco-awareness emerged as meaningful correlates of dietary adherence, whereas components of psychological distress showed consistently negative associations. Taken together, these findings suggest that environmental motivation and psychological well-being play intertwined roles in shaping adherence to healthy dietary patterns.

### 4.1 Relationship between psychological and environmental variables

Our correlation and regression analyses indicated that eco-anxiety was positively associated with depression, anxiety, and stress levels. The American Psychological Association (APA) defines eco-anxiety as a chronic fear of environmental catastrophe, noting that it can range from mild stress to clinical conditions such as depression, anxiety, and post-traumatic stress disorder (Clayton et al., 2017). Recent systematic reviews also support the association between eco-anxiety and these components of psychological distress. They further highlight that eco-anxiety may represent a potential mental health risk factor, particularly when coping mechanisms are insufficient (Boluda-Verdú et al., 2022; Cosh et al., 2024; White et al., 2023).

In a large-scale study conducted across 32 countries, eco-anxiety was found to be negatively associated with mental well-being in 31 countries, while positive associations between eco-anxiety and pro-environmental behaviors were observed in 24 countries (Ogunbode et al., 2022). In contrast, our findings showed no significant association between eco-anxiety and eco-awareness intended to reduce ecological footprint. This may align with the authors’ interpretation in the study, suggesting that the relationship between climate anxiety and pro-environmental actions may be influenced by multiple factors, ranging from individualism to national prosperity (Ogunbode et al., 2022). Similarly, a national study conducted with adults also found no significant association between these two variables. This finding suggests that the influence of eco-anxiety on attitudes and behavioral outcomes may be context-dependent and may operate through indirect mechanisms rather than exerting a direct effect (Bayram and Ozturkcan, 2025). However, this seemingly counterintuitive finding can be better understood by considering the nuanced distinction between the two constructs: eco-anxiety reflects an emotional response to environmental threat, whereas eco-awareness represents a knowledge -based cognitive process related to reducing environmental impact (Yildirim et al., 2025).

Our findings further indicated that higher levels of anxiety, as a component of psychological distress, were negatively associated with eco-awareness. In other words, lower anxiety levels were linked to greater engagement in pro-environmental behaviors. Supporting this, a previous study reported that having a stronger connection to nature and engaging with it more consciously was associated with better mental health outcomes (Chang et al., 2024). A study among adolescents also showed that pro-environmental behaviors may enhance psychological well-being. These behaviors may help individuals gain a more positive position within society and foster a sense of contributing to the improvement of the world, thereby adding meaning to life (Bartolo et al., 2023). Although this aspect does not directly reflect the primary aim of our study, it provides a valuable basis for a more comprehensive understanding of our secondary objectives.

### 4.2 Relationship between dietary adherence and ecological variables

It is possible to distinguish between pathological and adaptive forms of eco-anxiety, and this differentiation is important for interpreting our findings. Eco-anxiety reflects a negative state; however, it should not always be perceived in this way. The pathological form refers to anxiety that disrupts functioning and may reach clinical levels such as depression or anxiety disorders, whereas the adaptive form represents a type of anxiety that increases awareness, encourages problem-solving, and triggers adaptive coping mechanisms (Dodds, 2021). In other words, rather than leading to passivity, eco-anxiety may transform into active pro-environmental behaviors, which can have a protective effect on mental health (Boluda-Verdú et al., 2022) and, beyond that, promote adherence to healthy and sustainable dietary patterns. Our findings indicated that higher adherence to both the Mediterranean and DASH diets was associated with higher levels of eco-anxiety and eco-awareness. Regression analyses showed that only eco-anxiety was significantly associated with Mediterranean diet adherence, whereas both eco-anxiety and eco-awareness were significant correlates of DASH adherence.

This study’s sampling method may have resulted in a more educated, urban, and environmentally conscious sample, which may partly explain why eco-awareness was associated with DASH adherence but not with MEDAS adherence. The DASH diet is often perceived as a more structured and clinically oriented model, aligning more closely with individuals who make information-based decisions, whereas the Mediterranean diet is viewed as a more cultural and habit-driven pattern, which may lead to a weaker association with eco-awareness.

Previous studies have shown that individuals with higher levels of eco-anxiety tend to exhibit greater adherence to the Mediterranean diet compared to those with lower levels (Memiç-İnan et al., 2025), and that eco-anxiety is positively associated with both adherence to the Mediterranean diet and climate-friendly food choices. Furthermore, higher adherence to the Mediterranean diet has been associated with greater eco-awareness and pro-environmental behaviors (Kocaadam-Bozkurt and Bozkurt, 2023), findings that have also been supported by linear regression analyses (Kabasakal Cetin et al., 2025). In addition, climate change awareness has been identified as a factor positively associated with sustainable dietary patterns such as the Mediterranean diet (Metin et al., 2024), highlighting the relationship between being conscious of climate change and adopting climate-friendly dietary behaviors (Jürkenbeck et al., 2021; Korkala et al., 2014). Another study also reported that individuals with higher adherence to the Mediterranean diet were more likely to choose environmentally labeled foods (Yassıbaş and Bölükbaşı, 2023).

### 4.3 Relationship between dietary adherence and psychological variables

Our findings indicated that higher adherence to the Mediterranean diet was significantly associated with lower levels of depression and anxiety. Regression analyses showed that each component of psychological distress—depression, anxiety, and/or stress—was negatively associated with adherence to these diets. Previous studies have similarly reported negative associations between adherence to the Mediterranean diet and anxiety, stress, or depression (Houminer-Klepar and Dopelt, 2025; El Mikkawi et al., 2024; Ünal and Özenoğlu, 2025). Among the components of psychological distress, depression has been the most extensively examined in this context. A 20-year cohort study reported that each one-unit increase in adherence to the Mediterranean diet was associated with a 5% lower risk of depression (Yin et al., 2021). In a cross-sectional study, binary logistic regression analyses showed that moderate and high adherence to the Mediterranean diet were associated with reduced odds of depression even after adjusting for potential confounders, whereas no significant associations were observed for anxiety or stress (Jiménez-López et al., 2024). A large-scale study found that each one-point increase in the MEDAS score among individuals with obesity was associated with a 7% lower risk of anxiety and depression (Menichetti et al., 2025). Moreover, a systematic review has provided consistent evidence linking adherence to the Mediterranean diet with lower incidence rates of depression (Eliby et al., 2023). This association may be partly explained by the Mediterranean diet’s antioxidant and anti-inflammatory properties. The diet may reduce systemic inflammation, mitigate oxidative stress, regulate gut microbiota, support brain-derived neurotrophic factor expression, and modulate tryptophan metabolism and the stress response. Taken together, these mechanisms may offer a biological framework for understanding the associations between adherence to the Mediterranean diet and the underlying pathophysiological processes related to psychological distress (Yin et al., 2021; Menichetti et al., 2025).

Moreover, when various demographic and health-related confounding variables are controlled for, the negative association between adherence to the Mediterranean diet and anxiety remains statistically significant, whereas its association with depression becomes non-significant (Houminer-Klepar and Dopelt, 2025; Shiraseb et al., 2023). Another study reported that adherence to the Mediterranean diet was not significantly associated with depression levels but showed significant negative associations with anxiety and stress (Allcock et al., 2024). On the other hand, some findings indicate that adherence to the DASH diet is not associated with anxiety but is related to lower levels of severe depression and stress. Although adherence to the DASH diet has been suggested to offer mental health benefits, the existing findings remain inconsistent and sometimes contradictory (Shiraseb et al., 2023; Tan et al., 2023). Consistent with this pattern, our study found that while no significant correlations emerged between DASH adherence and psychological distress, the regression model indicated that—within our sample—DASH adherence was negatively associated with depression and anxiety, whereas stress did not appear as a significant correlate.

A recent comprehensive systematic review examined the relationships between various dietary patterns and psychological distress and consistently reported that the Mediterranean diet has the strongest and most robust evidence for protective effects on depression and anxiety. In contrast, findings related to the DASH diet were more limited, heterogeneous, and supported by weaker levels of evidence (Eliby et al., 2023). The Mediterranean and DASH diets may have been examined within different scientific contexts, particularly with respect to their potential associations with psychological well-being. Research on the Mediterranean diet has extensively explored bioactive components such as polyphenols, omega-3 fatty acids, and antioxidants, as well as their possible neurobiological implications. In contrast, the DASH diet was originally developed to address hypertension and cardiometabolic health (Yildirim et al., 2025), and therefore, investigations into its psychological effects and associated nutrient pathways have been comparatively less prominent. These differences in research focus may help explain the weaker and less consistent evidence linking the DASH diet to psychological distress (Koelman et al., 2022). Additionally, the DASH diet is not as widely recognized by the general population as the Mediterranean diet, which may influence participants’ interpretation of questionnaire items and the accuracy of self-reported adherence. Accordingly, the weaker associations observed in our study between DASH adherence and depression and anxiety—and the absence of a significant relationship with stress—are consistent with the existing literature and the fundamental compositional differences between these two dietary patterns.

### 4.4 The dual role of eco-anxiety in dietary adherence

Mediation analyses indicated that eco-anxiety showed direct positive associations with adherence to the Mediterranean and DASH diets, whereas indirect pathways involving psychological distress components—depression, anxiety, or stress—revealed negative associations. In other words, although eco-anxiety was generally positively associated with healthy dietary adherence, the indirect pathways related to psychological distress suggested that relationships in different directions may also be observed within this overall pattern.

Individuals experiencing psychological distress may engage in daily decision-making strategies aimed at avoiding perceived threats, which may be negatively associated with their ability to maintain adaptive health behaviors. Such individuals may prioritize avoiding potential negative outcomes over obtaining possible benefits. Furthermore, psychological distress may weaken interactions with neural systems involved in adjusting maladaptive decision-making processes (Hartley and Phelps, 2012). Psychological distress has also been associated with impaired decision-making and a stronger tendency toward habit-based choices. This pattern resembles what is observed in behavioral addictions such as eating disorders (Brydevall et al., 2025). Specifically, it may be associated with increased impulsive eating behaviors and higher consumption of high-calorie, fatty, sugary, fast-food, and ultra-processed products, which in turn may be linked to lower adherence to healthy dietary patterns (Christofaro et al., 2022).

Although psychological distress is generally perceived as a negative condition, eco-anxiety may function as a motivational force that encourages individuals to engage in climate-related behaviors and may represent a protective emotional response aimed at safeguarding the planet (Clayton, 2020; Sampaio et al., 2023). Experiences related to climate change have been associated not only with psychological distress but also with positive coping strategies. These strategies are linked to greater participation in environmental actions. These findings suggest that eco-anxiety may represent a functional response (Clayton and Karazsia, 2020). In this context, several cognitive and behavioral mechanisms may help explain why psychological distress could weaken the potential motivational component of eco-anxiety. First, according to Cognitive Load Theory, heightened psychological distress may increase cognitive burden, reducing an individual’s capacity to process information and translate intentions into action (Kong et al., 2025). Second, frameworks such as Self-Regulation Theory and models of executive functioning suggest that conditions like depression and anxiety may impair self-control, planning, and goal-directed behavior, making it more difficult to maintain long-term health behaviors such as healthy dietary adherence (Billore et al., 2023). Third, habit-based decision-making models propose that psychological distress shifts decision processes toward more automatic, impulsive, and short-term reward–seeking patterns (Sarmiento et al., 2024). This pattern can also be interpreted through frameworks such as Protection Motivation Theory (Balla and Hagger, 2025; Mokhtari Karchegani and Savari, 2025) and Threat Appraisal Theory (Krok et al., 2023), which propose that the translation of perceived threat into adaptive action depends on cognitive appraisals and coping resources; when these resources are overwhelmed by psychological distress, motivated intentions—such as those driven by eco-anxiety—may fail to convert into sustained health behaviors. Taken together, these theoretical mechanisms provide a plausible explanation for the negative indirect pathways observed in the mediation models, indicating that psychological distress may shape the direction and strength of associations between eco-anxiety and dietary adherence. This pattern is consistent with partial mediation, in which the indirect effects attenuate—but do not eliminate—the direct association, suggesting that a motivational component of eco-anxiety remains independent of psychological distress.

Given the cross-sectional nature of the study, the associations identified in the mediation models should not be interpreted as evidence of causal pathways. Although the models statistically represent the hypothesis that eco-anxiety may be associated with greater psychological distress, which in turn relates to dietary adherence, alternative explanations are equally plausible. For instance, individuals experiencing higher levels of psychological distress may become more sensitive to environmental threats, resulting in heightened eco-anxiety rather than the reverse. It is also possible that unhealthy dietary habits contribute to the development or worsening of psychological distress, indirectly shaping environmental concerns. More broadly, a general vulnerability to stress may underlie both eco-anxiety and diet-related behaviors. Therefore, the observed associations should be understood as bidirectional or multidirectional possibilities rather than a single linear pathway, reinforcing the need for longitudinal and experimental research to clarify temporal order and causal mechanisms.

### 4.5 Study limitations

This study provides valuable insights; however, several limitations should be acknowledged. First, the cross-sectional design restricts causal interpretation. Although the models were structured to examine pathways from eco-anxiety to psychological distress and dietary adherence, alternative directions are equally plausible. For example, general psychological distress may heighten eco-anxiety, or broader lifestyle-related factors may influence both psychological state and dietary behaviors. Therefore, the relationships identified in this study should be interpreted as associative rather than causal.

Second, the use of online convenience sampling limits the generalizability of the findings. Participation was restricted to individuals with internet access, which may have resulted in a sample that is more educated, urban, and potentially more environmentally aware than the general Turkish population. Such a sampling structure may have influenced the observed levels of eco-anxiety or strengthened its associations with dietary adherence. Additionally, participants’ baseline levels of environmental sensitivity could not be controlled, introducing a further source of potential bias.

Third, all variables—including anthropometric measurements and dietary adherence—were obtained through self-report. Self-reported height and weight are susceptible to social desirability and recall biases, which may reduce accuracy, particularly for BMI estimates. The lack of objective verification (e.g., measured anthropometrics) may have allowed implausible values or misreporting to persist despite screening procedures. Consequently, measurement error may have attenuated or inflated certain associations.

Finally, the online survey format excluded individuals with limited digital access or digital literacy, potentially reducing the representativeness of the sample. This limitation should be considered when interpreting the study findings.

## 5 Conclusion

This study examined the direct and indirect effects of eco-anxiety on adherence to the Mediterranean and DASH diets, with psychological distress as a mediator. The findings suggest that eco-anxiety may play a dual role: directly increasing diet adherence, while indirectly reducing it through psychological distress. This highlights the importance of considering the interplay between environmental concerns and mental health in shaping dietary behaviors.

This study suggests that environmental concerns and psychological factors may be associated with healthy and sustainable dietary behaviors. Findings indicate that while eco-anxiety can act as a motivating factor enhancing dietary adherence, psychological distress may weaken this relationship. Therefore, interventions promoting sustainable dietary practices may benefit from considering both environmental sensitivity and mental health support. Nevertheless, results should be interpreted with caution given the study’s limitations.

Future research should employ longitudinal and cross-cultural designs to test the directionality and generalizability of these associations. At the applied level, initiatives promoting sustainable diets may be more effective if they integrate components that address both environmental awareness and mental health support.

## Data Availability

The raw data supporting the conclusions of this article will be made available by the authors, without undue reservation.
